# Cytokine Signature Associated With Disease Severity in COVID-19

**DOI:** 10.3389/fimmu.2021.681516

**Published:** 2021-08-20

**Authors:** Jing Guo, Shuting Wang, He Xia, Ding Shi, Yu Chen, Shufa Zheng, Yanfei Chen, Hainv Gao, Feifei Guo, Zhongkang Ji, Chenjie Huang, Rui Luo, Yan Zhang, Jian Zuo, Yunbo Chen, Yan Xu, Jiafeng Xia, Chunxia Zhu, Xiaowei Xu, Yunqing Qiu, Jifang Sheng, Kaijin Xu, Lanjuan Li

**Affiliations:** ^1^State Key Laboratory for Diagnosis and Treatment of Infectious Diseases, National Clinical Research Center for Infectious Diseases, Collaborative Innovation Center for Diagnosis and Treatment of Infectious Diseases, Department of Infectious Diseases, The First Affiliated Hospital, College of Medicine, Zhejiang University, Hangzhou, China; ^2^The Shulan (Hangzhou) Hospital, Affiliated to Shulan International Medical College, Zhejiang Shuren University, Hangzhou, China

**Keywords:** COVID-19, SARS-CoV-2, hypercytokinaemia, biomarker, disease severity

## Abstract

Coronavirus disease 2019 (COVID-19) broke out and then became a global epidemic at the end of 2019. With the increasing number of deaths, early identification of disease severity and interpretation of pathogenesis are very important. Aiming to identify biomarkers for disease severity and progression of COVID-19, 75 COVID-19 patients, 34 healthy controls and 23 patients with pandemic influenza A(H1N1) were recruited in this study. Using liquid chip technology, 48 cytokines and chemokines were examined, among which 33 were significantly elevated in COVID-19 patients compared with healthy controls. HGF and IL-1β were strongly associated with APACHE II score in the first week after disease onset. IP-10, HGF and IL-10 were correlated positively with virus titers. Cytokines were significantly correlated with creatinine, troponin I, international normalized ratio and procalcitonin within two weeks after disease onset. Univariate analyses were carried out, and 6 cytokines including G-CSF, HGF, IL-10, IL-18, M-CSF and SCGF-β were found to be associated with the severity of COVID-19. 11 kinds of cytokines could predict the severity of COVID-19, among which IP-10 and M-CSF were excellent predictors for disease severity. In conclusion, the levels of cytokines in COVID-19 were significantly correlated with the severity of the disease in the early stage, and serum cytokines could be used as warning indicators of the severity and progression of COVID-19. Early stratification of disease and intervention to reduce hypercytokinaemia may improve the prognosis of COVID-19 patients.

## Introduction

Coronavirus disease 2019 (COVID-19) is a novel infectious disease caused by severe acute respiratory syndrome coronavirus 2 (SARS-CoV-2) that has rapidly spread throughout the world (1, 2). As of 16 June 2021, there have been 176,156,662 confirmed cases of COVID-19 worldwide, including 3,815,486 deaths, reported to WHO. Although a total of 2,310,082,345 vaccine doses have been administered, SARS-CoV-2 mutates rapidly, so the number of confirmed coronavirus cases and the proportion of deaths are still increasing.

With no effective treatment directly targeting SARS-CoV-2, COVID-19 is associated with a fatality rate of around 1-3%, which is commonly linked to the development of acute respiratory distress syndrome (ARDS) (3, 4). ARDS is a life-threatening complication as the alveolar-capillary barrier is compromised and fluid leaks into the lungs, which is the leading cause of mortality for COVID-19 patients (5). Hypercytokinemia, which is also called cytokine storm, was found in SARS-CoV-2 infection, and thought to contribute to acute lung injury and development of ARDS (6–9). Pathological changes consistent with cytokine storms have been observed in COVID-19 patients, particularly in severe or critically ill cases (10, 11).

Our previous study revealed that immunological disorders play an important role in the progression of COVID-19 (12, 13). The specific pathogenesis of cytokine storm is still unclear. The key to reduce the mortality of COVID-19 is to clarify the pathogenesis, explore specific therapeutic targets, and screen early targets for severe disease. Here, we investigated the serum cytokine in COVID-19 patients and attempted to find accurate markers for predicting fatal outcomes and reveal the immune mechanism related to COVID-19.

## Methods

### Clinical Specimens

Hospitalized COVID-19 patients were admitted between January 2020 and March 2020. Hospitalized pandemic influenza A (H1N1) patients and healthy volunteers were enrolled at the same time. COVID-19 and H1N1 infections were confirmed in the laboratory with protocols reported previously (14, 15). The classification of COVID-19 subtype was based on the eighth edition of Diagnostic and Treatment Protocol for COVID-19 in China. Mild-type COVID-19 cases included nonpneumonia and mild pneumonia. The severe type manifested as dyspnoea, respiratory rate ≥ 30 breaths per minute, blood oxygen saturation ≤ 93%, partial pressure of oxygen (PaO_2_)/fraction of inspired oxygen (FiO_2_) ratio < 300, and/or lung infiltrates > 50% within 24–48 hours. Critical cases exhibited respiratory failure, septic shock, and/or multiple organ dysfunction/failure. Patients with HIV, tumor, organ transplant status, pregnancy status and autoimmune diseases are excluded from this study. Finally we enrolled a total of 75 COVID-19 patients, including 28 mild cases, 30 severe cases and 17 critical cases.

We also recruited 23 H1N1 patients. The control group consisted of 34 healthy individuals who visited the First Affiliated Hospital of Zhejiang University for routine health examinations. All healthy subjects had normal liver biochemistry tests and Chest X-ray without evidence of cardiovascular diseases, liver diseases, diabetes or other diseases. A summary of the clinical information of the patients is shown in **Table 1** and **Supplementary Table 1**.

**Table 1:** Clinical characteristics of subjects including COVID-19 patients, H1N1 patients and healthy controls in this study.

Variable	COVID-19 (n=75)	H1N1 (n=23)	Healthy control (n=34)
Gender, male, n (%)	46 (61.3)	9 (39.1)	20 (58.8)
Age group–n (%)			
≤65	61 (81.3)	16 (69.6)	32 (94.1)
>65	14 (18.7)	7 (30.43)	2 (5.9)
Median	53	55	55
IQR	39-62.5	39-67	49.8-59
**Selected presenting signs and symptoms**			** **
Fever, n (%)	66 (88)	8 (34.8)^#^	/
Cough, n (%)	57 (76)	19 (82.6)	/
Expectoration, n (%)	34 (45.3)	14 (60.9)	/
Diarrhea, n (%)	9 (12)	0	/
**Blood routine examination**	** **	** **	
White blood cells, 10^9^/L–median (IQR)	5.9 (3.9-8.9)	7.3 (4.8-9.6)	5.6 (4.6-6.9)
Hemoglobin, g/L–median (IQR)	1347 (120-147.5)	135 (107-148)	145 (133.75-156.25)*
Platelet, 10^9^/L–median (IQR)	191 (162-252.5)	170 (134-244)	235 (199.5-269.25)*
Neurtophil , 10^9^/L–median (IQR)	4.35 (2.6-7.5)	5.9 (3.4-7.8)	3.10 (2.34-3.69)*
Lymphocyte, 10^9^/L–median (IQR)	0.8 (0.5-1.2)	0.9 (0.6-1.4)	1.75 (1.38-2.19)*
**Coagulation function**			
D-Dimer, ug/L FEU–median (IQR)	375 (197.3-757)	209 (131.75-575.25)	/
**Biochemical examination**			
ALT, U/L–median (IQR)	22 (14.5-34)	23 (16.5-44.5)	16.5 (13-21.8)*
AST, U/L–median (IQR)	22 (18-34)	23 (21-38)	19 (15.5-21)*
LDH, U/L–median (IQR)	247 (207.5-344)	258 (221-279)	/
CRP, mg/L–median (IQR)	17.7 (6.9-49.2)	27.2 (10.53-66.2)	/
Cr, umol/L–median (IQR)	75 (61-88.5)	66 (59-89)	68.5 (60-79.3)
Serum ferritin, ng/mL–median (IQR)	520.5 (255.8-982.3)	289.2 (194.2-484.2)	/
**APACHEⅡscore–median (range)**	5 (0-19)	5 (1-15)	/
**Underlying disease**			
Cardiovascular diseases, n (%)	4 (5.3)	2 (8.7)	/
Respiratory disorders, n (%)	3 (4)	5 (21.7)^#^	/
Liver diseases, n (%)	8 (10.7)	2 (8.7)	/
Diabetes, n (%)	10 (13.3)	1 (4.3)	/
Hypertension, n (%)	25 (33.3)	5 (21.7)	/
**Treatment**			
Antibiotic therapy, n (%)	28 (37.3)	6 (26)	/
Glucocorticoid therapy, n (%)	57 (76)	0 (0)^#^	/
Antiviral therapy, n (%)	75 (100)	23 (100)	/
Mechanical ventilation, n (%)	6 (8)	0 (0)	/
ECMO, n (%)	5 (66.7)	0 (0)	/
**First detection time points of cytokine**			
within 1st week after COVID-19 onset, n (%)	9 (12%)	/	/
within 2nd week after COVID-19 onset, n (%)	46 (61%)	/	/
2 weeks later after COVID-19 onset, n (%)	20 (27%)	/	/

Data are displayed as n (%) or median (IQR). N is the total number of patients with available data. COVID‐19, coronaviru disease 2019; IQR, Interquartile range; ALT, alanine aminotransferase; AST, aspartate aminotransferase; LDH, lactate dehydrogenase; CRP, C-reactive protein; Cr, creatinine; APACHE II, Acute Physiology and Chronic Health Evaluation II; ECMO, extracorporeal membrane oxygenation. *p < 0.05: COVID-19 vs healthy control; ^#^p < 0.05: COVID-19 vs H1N1.

This study was approved by the Institutional Review Board of the First Affiliated Hospital, School of Medicine, Zhejiang University (numbers IIT2020-136, numbers IIT20200148A), and written informed consent was obtained from all participants. The Declaration of Helsinki was strictly followed.

#### Data Extraction

Clinical and demographic data were retrieved from all participants electronic medical records. These data included age, gender, comorbidities, symptoms, the severity of illness scores at admission. APACHE II score is a severity of disease classification system, which based upon initial values of 12 routine physiologic measurements, age, and previous health status to provide a general measure of disease severity (16). Here we used to assess the severity of COVID-19 as previously reported (17, 18). Laboratory variables that were evaluated included levels of blood routine examination (white blood cells, hemoglobin, platelet, neutrophil and lymphocyte), coagulation function (D-Dimer), biochemical examination [alanine aminotransferase (ALT), aspartate aminotransferase (AST), lactate dehydrogenase (LDH), c-reactive protein (CRP), creatinine (Cr)] and serum ferritin. The reference values for the normal ranges of laboratory tests were in accordance with those used by the hospital laboratory.

Human pharyngeal swabs or sputum specimens and peripheral venous blood were collected at the earliest possible time point after hospitalization (9). Blood from healthy subjects was collected after physical examination. Serum samples were processed in the laboratory within 4 hours after collection and stored at −80°C until analysis. All biochemical indices were measured using an automatic analyzer (Hitachi 7600, Tokyo, Japan). Hematological parameters were analyzed using an automated hematology analyzer (Sysmex XN-9000). International normalized ratio (INR) was determined using the coagulation method with a Sysmex CS-2000i Analyser (Sysmex, Kobe, Japan) (19, 20).

#### Extraction of RNA and Real-Time RT-PCR

Viral infections were confirmed by real-time reverse-transcription polymerase chain reaction (PCR) with the RNeasy Mini Kit (QIAGEN, Germany) using sputum and throat swab specimens (8). If the data passed the quality controls, they were analysed.

### Cytokine and Chemokine Measurements

Multiplex immunoassays based on magnetic beads for selected serum biomarkers by a Bio-Plex Pro Human Cytokine Screening Test Kit (48-Plex #12007283, Bio-Rad), with a Bio-Plex 200 Suspension Array System (Bio-Rad, Hercules, CA) were processed in a BSL-2 Laboratory following the manufacturers’ instructions. Primary data were analysed using Bio-Plex Manager Software Version 6.1.1. Forty-eight cytokines that were quantified are listed as follows: Cutaneous T-cell attractant chemokine (CTACK); Eosinophil chemotactic protein (Eotaxin); basic fibroblast growth factor (FGF Basic); granulocyte colony-stimulating factor (G-CSF); granulocyte-macrophage colony-stimulating factor (GM-CSF); chemokine (C-X-C motif) ligand (CXCL) 1 (GRO-α); hepatocyte growth factor (HGF); interferon (IFN) alpha 2; IFN-γ; interleukin (IL) IL-1α; IL-1β; IL-1 receptor agonist (IL-1ra ); IL-2; IL-2ra; IL-3; IL-4; IL-5; IL-6; IL-7; IL-8; IL-9; IL-10; IL-12 p40 subunit (IL-12 (p40)); IL-12(p70); IL-13; IL-15; IL-16; IL-17; IL-18; Interferon-γ-inducible protein 10 (IP-10); leukemia inhibitory factor (LIF); monocyte chemoattractant protein (MCP) 1; MCP-3; macrophage colony-stimulating factor (M-CSF); Macrophage migration inhibitory factor (MIF); chemokines monokine induced by interferon (IFN)-γ (MIG); macrophage inflammatory protein (MIP) 1α; MIP-1β; beta-nerve growth factor (β-NGF); platelet-derived growth factor bb (PDGF-BB); regulated on activation, normal T cell expressed and secreted (RANTES); stem cell factor (SCF); stem cell growth factor beta (SCGF-β); stromal cell–derived factor 1a (SDF-1α); tumor necrosis factor -alpha (TNF-α); TNF-β; TNF-related apoptosis-inducing ligand (TRAIL); vascular endothelial growth factor (VEGF).

### Statistical Analysis

Statistical analyses were performed with SPSS (version 20.0, SPSS Inc, Chicago, IL, USA). The figures were generated by GraphPad Prism (version 8.0, La Jolla, CA, USA). Continuous variables were expressed as median and interquartile range (IQR) values. The Kolmogorov–Smirnov test was used to assess whether continuous data were normally distributed. For the data of continuous numerical variables that conform to normal distribution and have homogeneity of variance, we carried out variance analysis to determine whether differences in the levels of cytokines between groups were statistically significant. For the data that do not exhibiting a normal distribution, nonparametric test was used. The differences between rates were tested by chi-square test or Fisher exact test, if appropriate. Correlations between variables were evaluated using Spearman’s correlation test. Logistic regression and receiver operating curve (ROC) analyses were performed to determine the diagnostic value. In all analyses, a P value < 0.05 derived from a 2-tailed test was considered statistically significant. For multiple comparison, we used Bonferroni correction to adjust P value.

## Results

### Patient Information

The main demographic characteristics of enrolled COVID-19 patients (COVID-19 group), H1N1 patients (H1N1 group) and healthy controls (HC group) were similar. The median age of the COVID-19 patients was 53 years (IQR, 39-62.5 years), and 46 (61.3%) were men. The median age of the H1N1 patients was 55 years (IQR, 39–67 years), 9 (39.1%) were men. The median age of healthy controls were 55 years (IQR, 49.8-59 years), and 20 (58.8%) were men. There was no statistical difference in age and sex among the three groups.

In COVID-19 group, the most common comorbidities were hypertension (33.3%), diabetes (13.3%) and liver diseases (10.7%). The main symptoms of the COVID-19 patients were fever and cough. The most common comorbidities were hypertension (21.7%) and respiratory disorders (21.7%) for H1N1 patients, and the most common symptoms were cough and expectoration. Respiratory disorders were more frequent in H1N1 patients, whereas other comorbidities (cardiovascular diseases, liver diseases, diabetes and hypertension) were equally distributed across H1N1 group and COVID-19 group. At the time point which serum specimens are collected, 6 patients were hospitalized in the ICU ward and the rest were in general isolation ward. The patient was transferred to the general ward after the nucleic acid turned negative. 1 COVID-19 patient underwent lung transplantation after the nucleic acid test of SARS-CoV-2 turned to negative. The mortality rate within 28 days was 0 in both COVID-19 group and H1N1 group.

### Hypercytokinaemia in COVID-19 Patients

**Figure 1:**
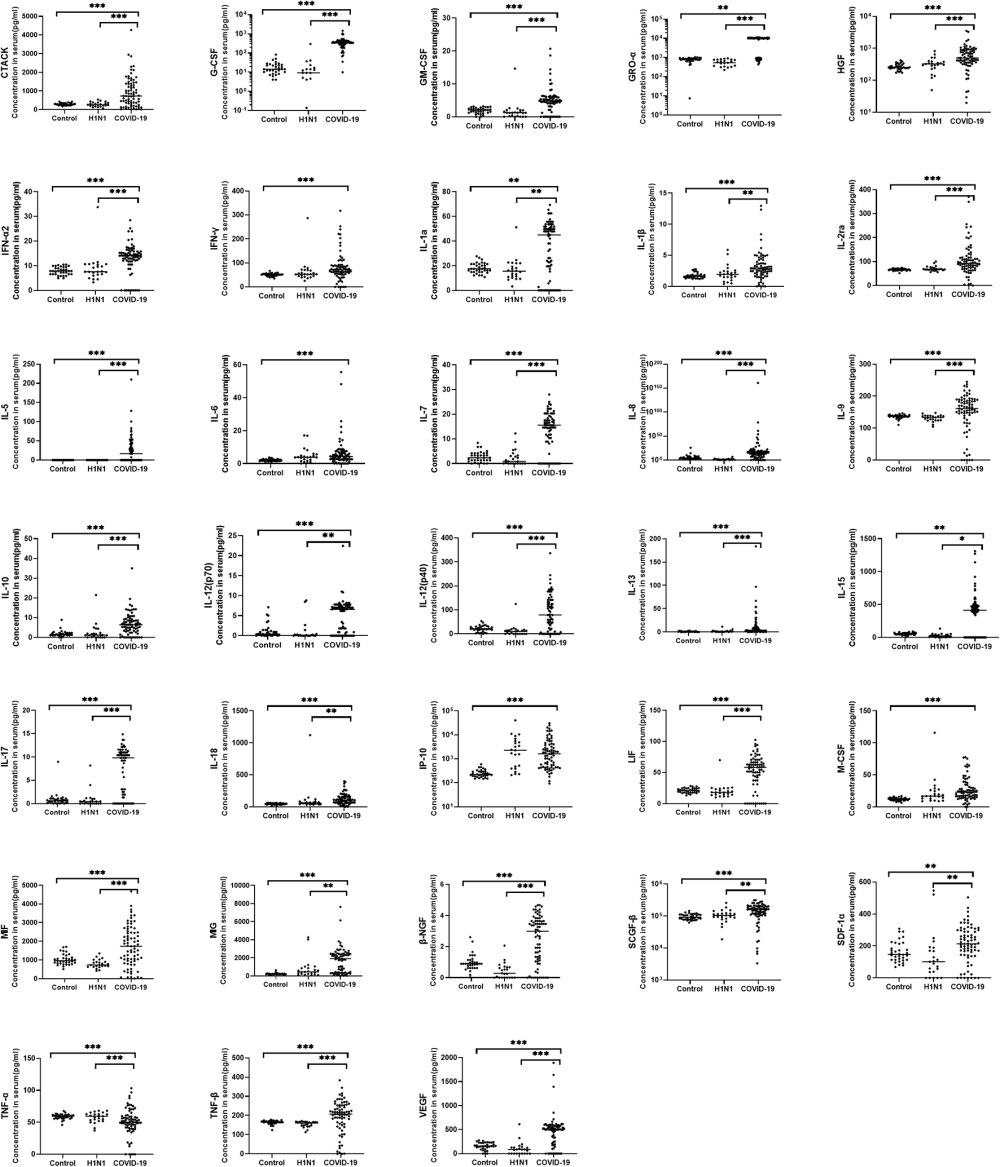
Comparison of serum cytokine concentrations between COVID-19 patients, H1N1 patients and healthy controls. Serum from COVID-19 patients (N=75), H1N1 patients (N=23) and healthy controls (N=34) were collected and 48 kinds of cytokines were measured. The horizontal line represents the median. Of the cytokine examined, 33 cytokines were significantly increased in COVID-19 patients when compared with the healthy controls, 29 cytokines were significantly increased in the COVID-19 group compare with H1N1 group. *P < 0.05, **P < 0.01, ***P < 0.001.

In this study, 48 cytokines and chemokines in the peripheral blood samples of the subjects were analysed. In general, hypercytokinaemia was observed in COVID-19 group compared with HC group and H1N1 group. 33 cytokines were significantly increased in the 75 COVID-19 patients compared with the HC group (**Figure 1**), including CTACK, G-CSF, GM-CSF, GRO-a, HGF, IFN-α2, IFN-γ, IL-1a, IL-1β, IL-2ra, IL-5, IL-6, IL-7, IL-8, IL-9, IL-10, IL-12(p70), IL-12(p40), IL-13, IL-15, IL-17, IL-18, IP-10, LIF, M-CSF, MIF, MIG, β-NGF, SCGF-β, SDF-1a, TNF-α, TNF-β and VEGF.

Compared with the H1N1 group, 29 cytokines were significantly increased in the COVID-19 group, including CTACK, G-CSF, GM-CSF, GRO-α, HGF, IFN-a2, IL-1α, IL-1β, IL-2ra, IL-5, IL-7, IL-8, IL-9, IL-10, IL-12(p70), IL-12(p40), IL-13, IL-15, IL-17, IL-18, LIF, MIF, MIG, β-NGF, SCGF-β, SDF-1α, TNF-α, TNF-β and VEGF.

### Correlation Between Levels of Cytokines and SARS-CoV-2 Viral Titer

**Figure 2:**
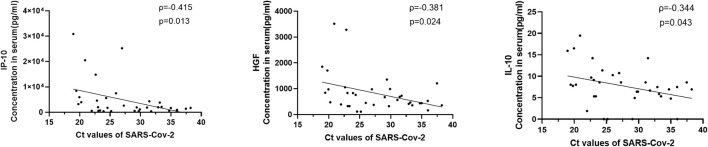
Correlations between serum cytokine level and virus titers in COVID-19 patients. The virus quantitative detection results were expressed by CT value for the SARS-CoV-2 gene by quantitative real-time reverse transcription-PCR as previously described. Viral loads from 35 patients within 2 weeks after disease onset were acquired. The levels of 3 cytokines, including IP-10, HGF and IL-10, were positively correlated with the virus titers.

Sputum or throat swab samples from COVID-19 patients were collected for detection of virus load. Sputum or throat swab samples from 35 patients were obtained on the same day blood was drawn within 2 weeks after disease onset; the virus titer was quantitatively detected, and the results are expressed by the CT value, as previously reported (21). Through correlation analysis between the virus CT value and 33 significantly elevated cytokines, the level of 3 cytokines, including IP-10 (ρ=-0.415, p=0.013), HGF (ρ=-0.381, p=0.024) and IL-10 (ρ=-0.344, p=0.043) were correlated positively with the virus titer (**Figure 2**).

### Association Between Levels of Cytokines and the Severity of COVID-19

**Figure 3:**
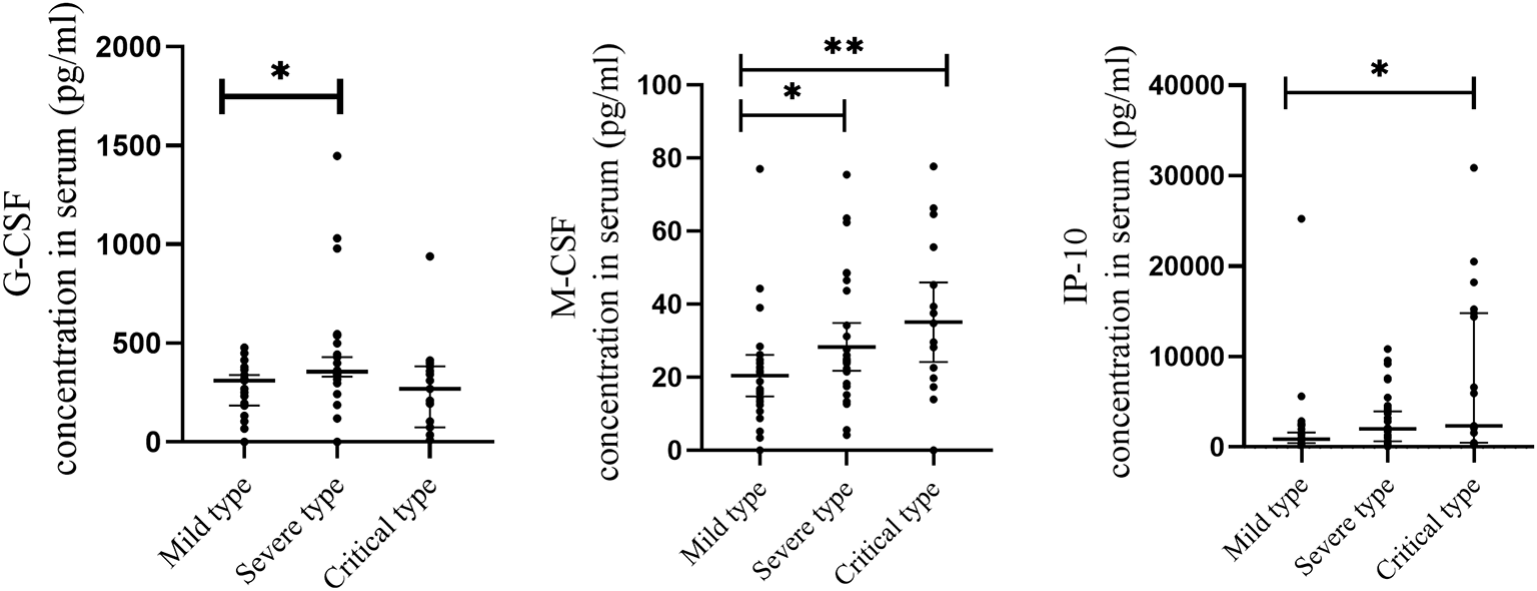
Comparison of serum cytokine concentrations between COVID-19 patients with different severity of illness. Serum samples from critically ill (N=17), severe (N=30), and mild (N=28) COVID-19 patients were collected at the earliest possible time point after hospitalization for assays measuring the concentrations of 48 cytokines. 2 cytokines with significantly increase in severe cases when compared with mild type, including G-CSF and M-CSF. 2 cytokines with significantly increase in critical cases when compared with mild type, including IP-10 and M-CSF. *P < .05, **P < .01.

To further determine whether the level of cytokines that significantly elevated are associated with the severity of COVID-19, cytokine levels were compared between mild, severe and critical types. As shown in **Figure 3**, when compared with the mild type, the level of 2 cytokines were significantly increased in severe cases, including G-CSF and M-CSF. When compared with the mild type, the level of 2 cytokines were significantly elevated in critical cases, including IP-10 and M-CSF.

At the same time, we analyzed the correlation between 33 significantly elevated cytokines and APACHE II score in 9 patients in the first week after disease onset. We found a significant positive correlation between the following cytokines and the severity of the COVID-19, which including IL-1β (ρ=0.77, p=0.015) and HGF (ρ=0.83, p= 0.006), while GRO-α (ρ=-0.68, p=0.046), IL-9 (ρ=-0.78, p=0.0139), TNF-α (ρ=-0.71, p=0.0325) and TNF-β (ρ=-0.86, p=0.0029) were negative correlated with disease severity (**Table 2**).

**Table 2:** Cytokine which highly related with APACHE II score in COVID-19.

	Week 1 (N = 9)		Week 2 (N = 46)	
Cytokine	Spearman	P value	Spearman	P value
GRO-a	-0.68	0.0460	-0.27	0.0740
IL-9	-0.78	0.0139	-0.04	0.7990
TNF-α	-0.71	0.0325	0.04	0.7970
TNF-β	-0.86	0.0029	-0.03	0.8330
IL-1β	0.77	0.0150	-0.05	0.7510
HGF	0.83	0.0060	0.11	0.4820

N is the total number of patients with available data. COVID‐19, coronavirus disease 2019; GRO-α, chemokine (C-X-C motif) ligand (CXCL) 1 ; IL-9, interleukin 9; TNF-α, tumor necrosis factor α ; TNF-β, tumor necrosis factor β; IL-1β, interleukin 1β; HGF, hepatocyte growth factor.

### Correlation Between Levels of Cytokines and Multisystem Function

To clarify the correlation between cytokines and clinical indicators, we analyzed the correlation between cytokines and creatinine, troponin I (TNI), procalcitonin (PCT), and international normalized ratio (INR) within two weeks after disease onset for significantly elevated cytokines (**Supplementary Table 2**). The correlation analysis showed that G-CSF (ρ=0.45, p=0.0007), HGF (ρ=0.43, p=0.0010), IL-1β (ρ=0.43, p=0.0010) and M-CSF (ρ=0.42, p=0.0015) were positively correlated with the level of Cr. GRO-a (ρ=-0.47, p=0.0006) and IL-17 (ρ=-0.43, p=0.0019) were negatively correlated with the level of TNI. IL-6 (ρ=0.41, p=0.0017) and IL-8 (ρ=0.42, p=0.0012) were positively correlated with the level of INR. IL-18 (ρ=0.46, p=0.0004) was positively correlated with the level of PCT.

### Independent Predictors of Cytokines and Their Diagnostic Value

Univariate analyses were carried out to identify prognostic factors for significantly elevated cytokines (**Table 3**). In this part, 55 patients within 2 weeks after COVID-19 onset were included. In univariate analysis, the level of G-CSF (OR = 1.0034, 95% CI 1.0000– 1.0067, p = 0.0475), HGF (OR = 1.0018, 95% CI 1.0001–1.0035, p = 0.0411), IL-10 (OR = 1.1930, 95% CI 1.0349– 1.3752, p = 0.0150), IL-18 (OR = 1.0087, 95% CI 1.0002– 1.0173, p = 0.0460), M-CSF (OR = 1.0541, 95% CI 1.0066– 1.1038, p = 0.0251) and SCGF-β (OR = 1.000, 95% CI 1.000– 1.000, p = 0.0271) were associated with the severity of COVID-19.

**Table 3:** Results of univariate analyse identifying independent cytokine associated with the severity of COVID-19.

	OR	95% confidence interval		p value
		upper limit	lower limit	
G-CSF	1.0034	1.0000	1.0067	0.0475
HGF	1.0018	1.0001	1.0035	0.0411
IL-10	1.1930	1.0349	1.3752	0.0150
IL-18	1.0087	1.0002	1.0173	0.0460
M-CSF	1.0541	1.0066	1.1038	0.0251
SCGF-β	1.0000	1.0000	1.0000	0.0271

COVID‐19, coronavirus disease 2019; OR, odds ratio; G-CSF, granulocyte colony-stimulating factor; HGF, hepatocyte growth factor; IL-10, interleukin 10; IL-18, interleukin 18; M-CSF, macrophage colony-stimulating factor; SCGF-β, stem cell growth factor beta.

ROC curve analysis was also performed to evaluate the relative efficiencies to predict the severity of the disease for significantly elevated cytokines. 55 patients within 2 weeks after COVID-19 onset were included in the analysis, as shown in **Table 4**. 11 kinds of cytokines could predict the prognosis of patients with severe and critical disease, including G-CSF, HGF, IL-6, IL-7, IL-8, IL-10, IL-18, IP-10, M-CSF, MIG and SCGF-β. Among which IP-10 (AUC: 0.74, 95%CI: 0.61-0.88, P =0.003) had the largest area under ROC curve, and IP-10 was also the most sensitive cytokine. M-CSF (AUC: 0.72, 95%CI: 0.59 - 0.86, P =0.006) was the most specific cytokine, which was also an excellent predictor for disease severity.

**Table 4:** Receiver operating characteristic (ROC) curve analysis by cytokines for COVID-19 patients to predicting severity of the disease.

Cytokine	Cutoff value	AUC	95% confidence interval		Sensitivity	Specificity	Youden Index	P
			lower limit	upper limit				
G-CSF	341.8	0.68	0.54	0.83	0.61	0.77	0.38	0.022
HGF	449.6	0.68	0.53	0.82	0.67	0.68	0.35	0.028
IL-6	6.9	0.68	0.53	0.82	0.42	0.91	0.33	0.027
IL-7	15.1	0.66	0.51	0.81	0.70	0.59	0.29	0.048
IL-8	16.8	0.67	0.52	0.81	0.58	0.82	0.39	0.037
IL-10	7.6	0.69	0.55	0.83	0.55	0.77	0.32	0.017
IL-18	101.9	0.67	0.52	0.81	0.64	0.64	0.27	0.036
IP-10	1693.0	0.74	0.61	0.88	0.73	0.73	0.45	0.003
M-CSF	29.1	0.72	0.59	0.86	0.42	0.95	0.38	0.006
MIG	2310.4	0.67	0.53	0.81	0.55	0.77	0.32	0.033
SCGF-β	192055.4	0.67	0.53	0.82	0.48	0.86	0.35	0.029

COVID‐19, coronavirus disease 2019; AUC, area under the curve; G-CSF, granulocyte colony-stimulating factor; HGF, hepatocyte growth factor; IL-6, interleukin 6; IL-7, interleukin 7; IL-8, interleukin 8; IL-10, interleukin 10; IL-18, interleukin 18; IP-10, Interferon-γ-inducible protein 10; M-CSF, macrophage colony-stimulating factor; MIG, chemokines monokine induced by interferon (IFN)-γ ; SCGF-β, stem cell growth factor beta.

## Discussion

COVID-19 remains a continuing threat globally due to the increasing number of deaths. Medical scientists and biologists around the world are still working to reduce morbidity and fatality. It is important to clarify the pathogenesis of the disease, the early warning indicators and the intervention targets of the disease (6, 7, 22). In our study, we revealed hypercytokinemia is present in patients with COVID-19, and significantly associated with the severity of early stage.

In our study, 33 cytokines were significantly elevated in COVID-19 patients compared with healthy controls. Both anti-inflammatory cytokines (such as IL-10 and IL-13) and pro-inflammatory cytokines (such as IL-1β, IL-6, IP-10, G-CSF, IL-8, IL-17 and IFN-γ) were significantly increased in COVID-19 patients, suggesting a serious immune disorder, which has been reported in other pathogenic coronaviruses (CoV) including Middle East respiratory syndrome coronavirus (MERS-CoV), severe acute respiratory syndrome coronavirus (SARS-CoV) (21, 23, 24). We compared the current literature on cytokines of COVID-19, and found that the research methods of the three studies were similar. Due to the different levels of disease severity, the results are not exactly the same. There are 38 (6), 27 (7) and 31 (22) cytokines increased in this three study compared with healthy controls, while among which 27(27/38, 71%), 22(22/27, 81.5%) and 24(24/30, 80%) cytokines were also increased in our study separately. Moreover, 14 cytokines were significantly increased in all 4 studies, including HGF, IL-1β, IL-2ra, IL-6, IL-7, IL-10, IL-13, IL-18, G-CSF, M-CSF, MIG, IP-10, IFN-a2 and IFN-γ. IP-10, also known as CXCL10, has been demonstrated that it can induce the chemotactic activity and migration of granulocyte, monocytes, macrophages and lymphocytes (25). A large number of studies on COVID-19 reported that IP-10 has been considered as an important biomarker of severe disease (6, 22, 25–28). IP-10-CXCR3 signaling seems to be a key factor in the pathological deterioration of SARS, H7N9 infection and ARDS (21, 23, 29). It has been reported that influenza infections had higher IP-10 concentrations than coronavirus, enterovirus or rhinovirus, and paramyxovirus (30). Our study found that there was no significant difference in the elevated level of IP-10 between patients with H1N1 and COVID-19. Therefore, we believe that IP-10 may play an important role in lung injury due to a variety of causes, rather than a specific marker of COVID-19. Antibody targeting IP-10 may be a promising strategy for the treatment of lung injury and ARDS.

IL-13 and IL-10 as anti-inflammatory cytokines were also significantly elevated in COVID-19 patients. A study that enrolled 548 patients found that high cytokine levels (IL-2R, IL-6, IL-10, and TNF-α) were significantly associated with severe COVID-19 on admission, whose findings correspond with our results (31). The appearance of elevated IL-10 may not play an effective protective role, but suggest a latent immune effort to control cytokine storm, which are unfortunately too late (32). Studies have revealed that IL-13 is a driver of COVID-19 severity (33), and could disrupts type 2 pneumocyte stem cell activity (34). While IL-13 neutralization results in reduced disease and lung hyaluronan deposition. The role of these anti-inflammatory factors remains to be further elucidated.

Among the 33 elevated cytokines, 29 cytokines in the COVID-19 group were higher than those in the H1N1 group, but there were no significant differences in IP-10, M-CSF, IFN-γ, and IL-6. This may indicate that these 4 cytokines may play an important role in the severity of viral respiratory diseases. Studies have reported differences in immunological characteristics between patients with H1N1 and COVID-19 (17). But our findings reveal more severe cytokine storms in COVID-19 compared with H1N1. The pathologic findings of the H1N1 patients are typical of alveolar pneumonia, which includes alveolar edema and inflammatory infiltrates in the lungs, preserving the integrity of the alveolar walls and the microstructure of the organs (17). And the inflammatory infiltrates observed in the lungs were composed of macrophages, polymorphonuclear cells, and scarce lymphocytes. SARS-CoV-2 induced intense and extensive inflammatory lung infiltrates, as well as thickness of alveolar walls, hemorrhages, and partial loss of the histological architecture of the lung, and macrophages is the primary inflammatory infiltrating cell in the lungs. The significantly elevated cytokine levels in peripheral blood were parallel to lung injury, further confirming the importance of cytokine storm in lung injury.

Many studies have focused on the correlation between viruses and the severity of diseases, but the research reports on the correlation between cytokines and viruses are limited. We found that the level of IP-10 was moderately positively correlated with viral titers, while the levels of HGF and IL-10 were weakly positively correlated with viral titers. Mathieu Blot’s studies found that the concentrations of IP-10 were elevated in the COVID-19 ARDS group, but without correlation between ELF IP-10 and viral load (26). Ying Chi has reported that the serum levels of MCP-1, G-CSF, and VEGF were weakly and positively correlated with viral titers (7). Yingxia Liu revealed 17 cytokines were linked to 2019-nCoV loads according to 25 samples from 12 COVID-19 patients (6). In our study, 3 elevated cytokines including IP-10, HGF and IL-10 were included in Yingxia Liu’s finding. These studies were characterized by a small number of cases, repeated sampling of the same patient and mild cases. Our results suggest the correlation between viruses and cytokines in the early stages of COVID-19. In comparison, our research is more complete and more convincing.

Statistical analysis showed that the cytokines of COVID-19 patients within 1 week after disease onset were correlated with the severity of the disease. The levels of HGF and IL-1β in the first week after disease onset were positively correlated with disease severity, while the levels of four cytokines (GRO-a, IL-9, TNF-α and TNF-β) were negatively correlated with APACHE II score. There was no such correlation in the second week after disease onset, indicating that hypercytokinemia in the early stage after disease onset was closely related to the severity of COVID-19, and that immune disorders might be the initial factor causing the severity of the disease. Whether GRO-a, IL-9, TNF-α and TNF-β play protective roles in COVID-19 has not been eliminated at present, which needs further confirmation by basic experiments. Similar to Chi Ying's findings, they reported most of the cytokines whose levels were associated with the severity of COVID-19 peaked at 6 to approximately 8 days (acute phase of disease) after onset (7). Therefore, we believe that early control of cytokine storm can improve the progression of the disease. But once in the late stage, cytokine storm is no longer correlated with the severity of the disease, which has caused organ damage and secondary infection, and it is difficult to reverse the disease.

The progression of COVID-19 usually involves multiple organs damage. Correlation analysis of cytokines and clinical indicators showed that hypercytokinemia was closely related to cardiac function, renal function and coagulation function. It may even be associated with secondary infections. IL-6 and IL-8 were positively correlated with INR here. Lung-centric coagulopathy may play an important role in the pathophysiology in the severe COVID-19 patients (35). It's reported that IL-6 may contribute to this pathology by inducing coagulation cascades (36), and IL-8 also could activates coagulation, which may be possible therapeutic targets (37). A significant positive correlation was found between IL-18 and PCT in our study. Indeed IL-18 is a biomarker to differentiate sepsis and septic shock status (38). Serum IL-18 concentration was found to correlate with inflammatory markers and reflect COVID-19 severity, consistent with our findings (39). Therefore, blocking cytokine storm in time may play an important role in protecting organ function and even avoiding secondary infection. Several studies have attempted to treat cytokine storm with monoclonal antibodies, including anti-IL-6 and anti-IL-8 in small samples (40–42). Our previous study found that the use of artificial liver support system can clear the cytokine storm and reduce the mortality of COVID-19, which indirectly supports this theory (29).

We analyzed whether these cytokines could be used as predictors for the disease progression of COVID-19. Our subjects were divided into moderate group and severe /critically group, just as the study reported by Yang Yang, in whose study subjects were divided into two groups, including non-ARDS group (the moderate patients) and ARDS group (critically ill and severe patients). 6 cytokines, including IP-10, MCP-3, IL-1ra, M-CSF, HGF and IL-6 were found highly associated with disease severity and predict the progression of COVID-19, among which 4 cytokines are consistent with our study. While in our study, 11 cytokines were shown to have good predictive ability. The predictive power of IP-10 was the best, with the highest sensitivity. Cytokines including M-CSF, G-CSF, HGF, IL-6, IL-7, IL-8, IL-10, IL-18, MIG and SCGF-β also exhibited good predictive value.

There are some limitations in our study. First, this is a single-center retrospective study. Due to the timely control of the epidemic, the sample size was small, especially lack of longitudinal samples. In the future, more multi-center studies in other parts of our country, and even other countries with different levels of disease severity are needed to verify these results. Because correlation does not necessarily reflect any causation, studies in cell and animal models are needed for a comprehensive interpretation of the clinical results. Finally, serum isolated from peripheral blood may not fully reflect the immune response that occurs in infected tissues, another cytokine milieu including peripheral intracellular cytokines, and even in the organs such as lung, kidney, and heart are also need to be further investigated.

In this study, we revealed the correlation between the cytokine storm induced by SARS-CoV-2 and the severity of early disease. A profile of cytokines, including IP-10 and M-CSF can be used as biomarkers for the prediction of severity in the early stage of COVID-19. Blocking cytokine storm may improve multiple organ function and reduce mortality in COVID-19 patients. Thus our findings provide a theoretical basis that early blocking of cytokine storm plays an important role in the treatment of disease, and timely identification of severe disease is of great significance for the diagnosis and treatment of COVID-19.

## Data Availability Statement

The original contributions presented in the study are included in the article/**Supplementary Material**. Further inquiries can be directed to the corresponding author.

## Ethics Statement

The studies involving human participants were reviewed and approved by the Institutional Review Board of the First Affiliated Hospital, School of Medicine, Zhejiang University. The patients/participants provided their written informed consent to participate in this study. Written informed consent was obtained from the individual(s) for the publication of any potentially identifiable images or data included in this article.

## Author Contributions

LJL conceived the project and designed the experiments. DS, YC, SFZ, YFC, HNG, FFG, CJH, RL, YZ, YX and JZ collected clinical samples and specimen. JG, STW, HX, ZKJ, YBC, JFX, CXZ performed the experiments. JG analysed the data, wrote the initial draft with all authors providing critical feedback. XWX, YQQ, JFS and KJX contributed fruitful discussions and helpful ideas. LJL made critical revision of the manuscript. All authors contributed to the article and approved the submitted version.

## Funding

This work was funded by the National Nature Science Foundation of China (U20A20343) and the Zhejiang Provincial Natural Science Foundation of China (No. LED20H190001, 2020C03123 and LQ17H030002).

## Conflict of Interest

The authors declare that the research was conducted in the absence of any commercial or financial relationships that could be construed as a potential conflict of interest.

## Publisher’s Note

All claims expressed in this article are solely those of the authors and do not necessarily represent those of their affiliated organizations, or those of the publisher, the editors and the reviewers. Any product that may be evaluated in this article, or claim that may be made by its manufacturer, is not guaranteed or endorsed by the publisher.
